# WindowNet: Learnable Windows for Chest X-ray Classification

**DOI:** 10.3390/jimaging9120270

**Published:** 2023-12-06

**Authors:** Alessandro Wollek, Sardi Hyska, Bastian Sabel, Michael Ingrisch, Tobias Lasser

**Affiliations:** 1 Munich Institute of Biomedical Engineering, TUM School of Computation, Information, and Technology, Technical University of Munich, 80333 Munich, Germany; lasser@cit.tum.de; 2 Department of Radiology, University Hospital Ludwig-Maximilians-University, 81377 Munich, Germany; sardi.hyska@med.uni-muenchen.de (S.H.); michael.ingrisch@med.uni-muenchen.de (M.I.)

**Keywords:** windowing, chest X-ray, chest radiograph, bit depth, classification, deep learning

## Abstract

Public chest X-ray (CXR) data sets are commonly compressed to a lower bit depth to reduce their size, potentially hiding subtle diagnostic features. In contrast, radiologists apply a windowing operation to the uncompressed image to enhance such subtle features. While it has been shown that windowing improves classification performance on computed tomography (CT) images, the impact of such an operation on CXR classification performance remains unclear. In this study, we show that windowing strongly improves the CXR classification performance of machine learning models and propose WindowNet, a model that learns multiple optimal window settings. Our model achieved an average AUC score of 0.812 compared with the 0.759 score of a commonly used architecture without windowing capabilities on the MIMIC data set.

## 1. Introduction

To better differentiate subtle pathologies, chest X-rays (CXRs) are commonly acquired with a high bit depth. For example, the images in the MIMIC data set provide 12-bit gray values; see [1]. However, to reduce the file size and save bandwidth, these images are often compressed to a lower bit depth. The Chest X-ray 14 data set, for example, was reduced to 8-bit depth before publication [2].

**Figure 1:**
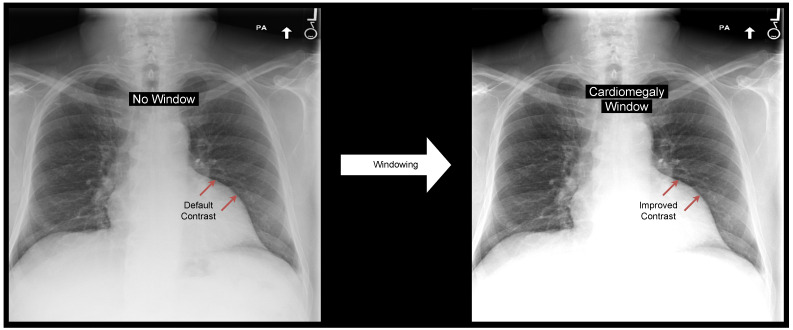
Applying a windowing operation enhances the contrast of particular structures of an image. For example, the depicted windowing operation improves cardiomegaly classification performance on the MIMIC data set.

Under optimal conditions, the human eye can differentiate between 700 and 900 shades of gray, or 9- to 10-bit depth [3]. Hence, radiologists cannot differentiate all 12-bit gray values when inspecting a chest X-ray. To better identify subtle contrasts, a windowing operation is applied to the image [4,5]: contrast is increased by limiting the range of gray tones (see Figure 1). These windowing operations can be specified by their center (level) and width.

In contrast to chest radiographs, gray values in computed tomography (CT) images are calibrated to represent a specific Hounsfield Unit (HU) [6]. For example, an HU value of −1000 corresponds to air, and 0 HU, to distilled water at standard pressure and temperature; bones range from 400 HU to 3000 HU [6]. To highlight the lung in a chest CT image, one could apply a window with a level of −600 HU and width of 1500 HU [7]. In other words, everything below −1350 HU is displayed as black, and everything above 150 HU, as white. Consequently, more distinct gray tone values can be used for the specified range, resulting in higher contrast.

For CT images, several studies showed that windowing improves the classification performance of deep neural networks [8,9,10,11]. For CXRs, no quantitative scale like Hounsfield Unit exists. Nevertheless, radiologists window CXRs for enhanced contrast during inspection. Furthermore, depending on the region of interest, they use different window settings. This observation leads to the following research questions: does windowing affect chest X-ray classification performance, and if so, can windowing improve it? To the best of our knowledge, so far, chest X-rays are commonly processed using a deep learning model without applying any windowing operation (for example, [12,13]). This study investigates the effect of windowing on chest X-ray classification and proposes a model, WindowNet, that learns optimal windowing settings.

Our contributions are as follows:

- We show that a higher bit depth (8-bit vs. 12-bit depth) improves chest X-ray classification performance.
- We demonstrate that applying a window to chest radiographs as a pre-processing step increases classification performance.
- We propose WindowNet, a chest X-ray classification model that learns optimal windowing settings.

## 2. Materials and Methods

### 2.1. Data Set

To investigate the importance of windowing in chest X-ray classification, we selected the MIMIC data set, as it is the only publicly available, large-scale chest X-ray data set with full bit depth [1]. The MIMIC data set provides chest radiographs in the original Digital Imaging and Communications in Medicine (DICOM) format with 12-bit-depth gray values, containing 377,110 frontal and lateral images from 65,379 patients. The images have been labeled according to the 14 CheXpert classes: atelectasis, cardiomegaly, consolidation, edema, enlarged cardiomediastinum, fracture, lung lesion, lung opacity, no finding, pleural effusion, pleural other, pneumonia, pneumothorax, and support devices [14]. In our experiments, we used the provided training, validation, and test splits. During pre-processing, the images were resized to 224×224 pixels.

### 2.2. Architectures

#### 2.2.1. Baseline

As a baseline model (baseline) for all experiments, we used DenseNet-121 [15] pre-trained on ImageNet [16], which is commonly used for chest X-ray classification [12,17,18]. For fine tuning, we replaced the classification layer with a 14-dimensional fully connected layer.

#### 2.2.2. WindowNet

**Figure 2:**
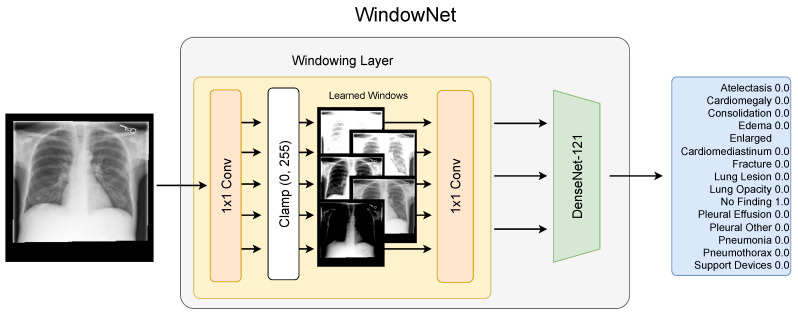
Optimal multi-window chest X-ray classification. Our proposed WindowNet architecture learns to optimize multiple windows for improved classification. The windowing operation is implemented as 1 × 1 convolution with clamping in the range (0, 255). The convolution weights are initialized with $\frac{255}{\mathrm{width}}$, and the bias, with $\frac{-255}{\mathrm{width}}\cdot \frac{\mathrm{level}-\mathrm{width}}{2}$, where width and level correspond to a channel’s initial windowing range.

To incorporate windowing into the model architecture, we extended the baseline architecture by prepending a windowing layer, as illustrated in Figure 2. In the following, we refer to this model as WindowNet.

We implemented the windowing operation as a 1×1 convolution operation with clamping, similar to [10]. This implementation of windowing utilizing convolutional kernels enables the model to learn and use multiple windows in parallel. As the pre-trained DenseNet-121 expects three input channels, we added an additional 1×1 convolution operation with three output channels after the windowing operation. Following the windowing layer, the images are scaled to the floating point range (0.0,255.0) and then normalized according to the ImageNet mean and standard deviation.

#### 2.2.3. Training

Both models were trained with binary cross-entropy loss, AdamW optimization with a learning rate of 1 × 10^−4^ [19], and a batch size of 32. During training, the learning rate was divided by a factor of 10 if the validation loss did not improve in three consecutive epochs. The training was stopped if the validation loss did not improve after 5 consecutive epochs. The final models were selected based on the checkpoint with the highest mean validation area under the receiver operating characteristic curve (AUC). Due to the exploratory nature of our research and the necessity for multiple comparisons, we refrain from providing *p*-values. Instead, we provide 95 % confidence intervals, which were computed using the non-parametric bootstrap method involving 10,000-fold resampling at the image level.

### 2.3. Experiments

#### 2.3.1. Eight-Bit vs. Twelve-Bit Depth

As applying a windowing operation in our experiments required a higher initial bit depth than that conventionally used for chest X-ray image classification, we first tested the effect of bit depth on classification performance. We trained the baseline model with 8-bit and 12-bit depth and compared mean and class-wise AUC scores. In both settings, no windowing operation was applied. However, the 12-bit images were still scaled to the floating point range (0.0,255.0). In both settings, the images were normalized according to the ImageNet mean and standard deviation.

#### 2.3.2. Single Fixed Window

To investigate whether windowing has an effect on classification performance, we trained the baseline model with a single fixed windowing operation applied to the 12-bit CXRs. After windowing, the images were scaled to have a maximum value of 255 and normalized according to the ImageNet mean and standard deviation.

For windowing, we used a fixed window level of 100 and levels ranging from 250 to 3500 in steps of 250. All levels were combined with fixed window widths of 500, 1000, 1500, 2000, and 3000. For evaluation, we compared the mean and class-wise AUCs of each model to the baseline with no windowing, i.e., a window level of 2048 and width of 4096.

#### 2.3.3. Trainable Multi-Windowing

To test if end-to-end optimized windows improve chest X-ray classification performance, we compared our proposed WindowNet to the baseline and a modified WindowNet without clamping in the windowing layer (No Windowing), i.e., a conventional 1×1 convolutional layer. Furthermore, we trained the “No Windowing” model with random contrast and brightness augmentations (Augmentations).

In our experiments, we used 14 windows based on the set of class-wise top 3 windows found during the single-window experiment and the additional full-range “window”. The selection was based on the validation results. We initialized the learnable windows with the resulting windows (level, width): (100, 3000), (1250, 1000), (1500, 3000), (1750, 2000), (1750, 3000), (2000, 2000), (2250, 2000), (2250, 3000), (2500, 2000), (2500, 3000), (2750, 3000), (3250, 1000), (750, 3000), and (2048, 4096). The comparison models, “No Windowing” and “Augmentations”, having a conventional 1×1 convolution operation, were default-initialized using Kaiming initialization [20].

### 2.4. Windowing

A windowing operation can be described by its center (window level) and width (window width). Formally, the windowing operation applied to a pixel value px can be defined as

(1) $$\begin{array}{cc} \mathrm{window}(px) & =\min(\max(px,L),U), \end{array}$$

(2) $$\begin{array}{cc} U & =WL+\frac{WW}{2}, \end{array}$$

(3) $$\begin{array}{cc} L & =WL-\frac{WW}{2}. \end{array}$$

where *U* is the upper limit and *L* is the lower limit of the window defined by window level *WL* and window width *WW*.

For efficient training, the windowing operation can be re-written using a clamped 1×1 convolution operation between 0.0 and 255. The weight matrix is initialized as $W=\frac{U}{WW}$, and the bias term, as $b=-\frac{U}{WW}L$, similar to [10]. More specifically, weights are initialized with $\frac{255}{WW}$, and the bias, with $\frac{-255}{WW}\cdot \frac{WL-WW}{2}$, where WW and WL correspond to a channel’s initial windowing range.

(4) $$\begin{array}{cc} \min\left(\max\left(Wx+b,0\right),U\right) & =\min\left(\max\left(\frac{U}{WW}x-\frac{U}{WW}L,0\right),U\right) \end{array}$$

(5) $$\begin{array}{cc} \; & =\min\left(\max\left(\frac{U}{WW}\left(x-L\right),0\right),U\right) \end{array}$$

(6) $$\begin{array}{cc} \; & =\min\left(\max(x-L,0),U\right) \end{array}$$

(7) $$\begin{array}{cc} \; & =\min(\max(x,L),U). \end{array}$$

To recover the window level and width after training, we compute

(8) $$\begin{array}{cc} WW & =\frac{U}{W}, \end{array}$$

(9) $$\begin{array}{cc} WL & =-\frac{b}{W}+\frac{WW}{2}. \end{array}$$

## 3. Results

### 3.1. Eight-Bit vs. Twelve-Bit Depth

The classification AUCs, when trained with 8-bit or 12-bit depth, are shown in Table 1. Training with 12-bit images improved the average classification performance compared with 8-bit images (0.772 vs. 0.759 AUC). Also, most (12/14) class-wise AUCs increased when training was conducted with a higher bit depth. The only exceptions were atelectasis and pleural effusion, where training with 8-bit images resulted in slightly higher AUCs, with 0.751 vs. 0.749 and 0.883 vs. 0.879, respectively.

### 3.2. Single Fixed Window

The results of training with fixed window chest X-rays are reported in Table 2. They demonstrate that windowing improved chest X-ray classification AUCs for most classes (12/14), except for fracture and pneumonia, with AUCs of 0.710 vs. 0.706 and 0.698 vs. 0.690, respectively. On average, the window with a level of 2500 and width of 3000 performed slightly better than the full-range one, with AUCs of 0.775 vs. 0.772. Across all windows, a window width of 3000 performed best with varying window levels.

A comparison of the four best-performing windows to the baseline is shown in Table 3. All five settings achieved similar average AUC scores. No single window performed consistently better across all classes, suggesting that multiple windows could overall improve the classification performance.

### 3.3. Trainable Multi-Windowing

The effect of learning multiple optimal windows using our proposed WindowNet is reported in Table 4, where it is compared to the baseline, and the WindowNet architecture without windowing (“No Windowing”) and random contrast and brightness augmentations (“Augmentations”). Overall, WindowNet performed considerably better, with an average AUC of 0.812 compared with 0.750 of the eight-bit baseline. When compared with a conventional 1×1 convolution operation in the WindowNet architecture (“No Windowing”), the results demonstrate the improvement of windowing, with average AUCs of 0.812 vs. 0.790. Training the WindowNet architecture with random contrast and brightness (“Augmentations”) improved the average AUC from 0.790 to 0.804. Training with random contrast and brightness augmentations improved the “No Windowing” results for some classes, similarly to learning multiple windows. For other classes, for example, pneumonia or pneumothorax, learning multiple windows further improved the classification AUC results from 0.727 to 0.750 and from 0.856 to 0.886, respectively.

For nearly all classes (12/14), our proposed WindowNet model achieved a higher AUC than the baseline trained with eight-bit images. For example, pneumothorax classification AUC improved from 0.802 to 0.886 with windowing. Only for the fracture and pleural other classes, the baseline model performed better, with AUCs of 0.664 vs. 0.615 and 0.823 vs. 0.793, respectively.

**Figure 3:**
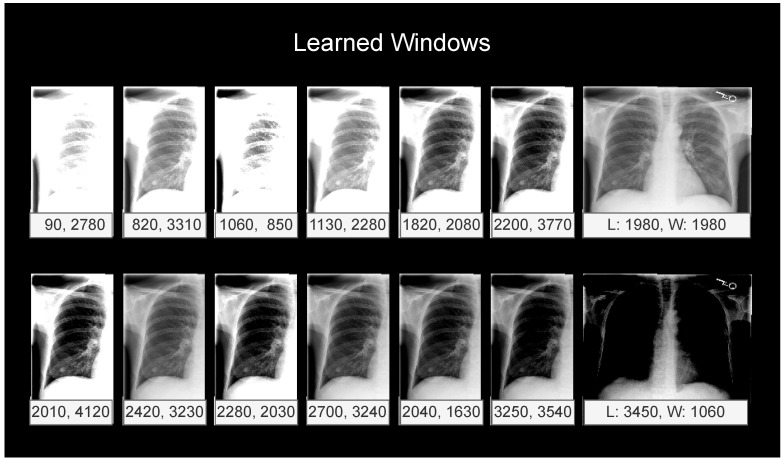
Windows learned during the training of WindowNet. For window initialization, the following window levels (L) and widths (W) were used (level, width): (100, 3000), (1250, 1000), (1500, 3000), (1750, 2000), (1750, 3000), (2000, 2000), (2250, 2000), (2250, 3000), (2500, 2000), (2500, 3000), (2750, 3000), (3250, 1000), (750, 3000), and no window (2048, 4096).

The windows learned after training are shown in Figure 3. The model learned a diverse set of windows, with levels from 90 to 3450 and widths from 850 to 4120.

## 4. Discussion

In this study, we investigated the importance of windowing, inspired by radiologists. Our results show that our proposed multi-windowing model, WindowNet, considerably outperformed a popular baseline architecture, with a mean AUC of 0.812 compared with 0.759 (see Table 4). As a necessary pre-condition, we also demonstrated that the common bit-depth reduction negatively affected classification performance (0.759 vs. 0.772 AUCs), as seen in Table 1. Based on these results, we recommend refraining from reducing image depth when storing or releasing data sets.

Similarly to related work in the CT domain [8,10,11], our results show that windowing is a useful pre-processing step for neural networks operating on chest X-rays. These findings are also in line with the observed manual windowing performed by radiologists in their daily practice. In addition, like radiologists apply multiple windows when inspecting a single image, no single window was better across classes, including not windowing at all (see Table 2).

When comparing our proposed WindowNet with the same architecture but without windowing, in other words, a conventional 1×1 convolution operation, our results show that the windowing operation is an important aspect of the architecture (see Table 4), even when accounting for training with random contrast and brightness augmentations. When inspecting the learned windows (see Figure 3), the windows converged to 14 different settings. This provides further evidence that multiple windows are important for classification performance.

While our study’s results are promising, limitations include the exploratory nature of the study and the evaluation on a data set from a single institution, due to the lack of other high-bit-depth public data sets. Further research is needed to show generalization to other data sets and institutions. Another limitation is that the model learns general windowing settings. In contrast, radiologists adapt the windowing settings based on the specific image. Future work could investigate an image-based window-setting prediction layer.

In conclusion, we believe our work offers an important contribution to the field of computer vision and radiology by demonstrating that multi-windowing strongly improves chest X-ray classification performance, as shown by our proposed model, WindowNet (https://gitlab.lrz.de/IP/windownet, accessed on 1 December 2023).

## Figures and Tables

**Table 1:** Effect of bit depth on chest X-ray classification performance. A higher bit depth improved AUC values for most (12/14) classes. Higher values are highlighted in bold. All AUC values with respective 95 % confidence intervals.

Finding	8-Bit Depth	12-Bit Depth
Atelectasis	**0.751** [0.736–0.767]	0.749 [0.733–0.764]
Cardiomegaly	0.770 [0.757–0.784]	**0.774** [0.760–0.788]
Consolidation	0.740 [0.715–0.765]	**0.742** [0.716–0.766]
Edema	0.831 [0.818–0.844]	**0.833** [0.820–0.846]
Enlarged cardiomediastinum	0.691 [0.656–0.726]	**0.701** [0.663–0.737]
Fracture	0.664 [0.624–0.705]	**0.710** [0.671–0.748]
Lung lesion	0.680 [0.644–0.716]	**0.682** [0.644–0.719]
Lung opacity	0.680 [0.665–0.695]	**0.690** [0.674–0.705]
No finding	0.789 [0.774–0.805]	**0.797** [0.781–0.811]
Pleural effusion	**0.883** [0.873–0.892]	0.879 [0.869–0.889]
Pleural other	0.823 [0.789–0.854]	**0.831** [0.799–0.860]
Pneumonia	0.659 [0.634–0.684]	**0.698** [0.674–0.721]
Pneumothorax	0.802 [0.766–0.836]	**0.828** [0.790–0.863]
Support devices	0.868 [0.857–0.879]	**0.888** [0.878–0.898]
Mean	0.759	**0.772**

**Table 2:** Effect of fixed windowing on chest X-ray classification AUCs. For each finding, the best-performing window and the baseline without windowing are reported. Higher AUCs values are highlighted in bold. Enlarged cardiom. = enlarged cardiomediastinum.

Finding	No Window	Best Fixed Window
Atelectasis	0.749 (2048, 4096)	**0.757** (2750, 3000)
Cardiomegaly	0.774 (2048, 4096)	**0.786** (1750, 3000)
Consolidation	0.742 (2048, 4096)	**0.744** (2500, 3000)
Edema	0.833 (2048, 4096)	**0.841** (1750, 3000)
Enlarged cardiom.	0.701 (2048, 4096)	**0.734** (2250, 3000)
Fracture	**0.710** (2048, 4096)	0.706 (1000, 3000)
Lung lesion	0.682 (2048, 4096)	**0.720** (2500, 3000)
Lung opacity	**0.690** (2048, 4096)	**0.690** (2250, 3000)
No finding	0.797 (2048, 4096)	**0.804** (2500, 3000)
Pleural effusion	0.879 (2048, 4096)	**0.888** (2500, 3000)
Pleural other	0.831 (2048, 4096)	**0.850** (2750, 3000)
Pneumonia	**0.698** (2048, 4096)	0.690 (1750, 3000)
Pneumothorax	0.828 (2048, 4096)	**0.832** (1750, 3000)
Support devices	0.888 (2048, 4096)	**0.889** (2750, 3000)
Mean	0.772 (2048, 4096)	**0.775** (2500, 3000)

**Table 3:** Best fixed single-window settings for chest X-ray classification found during grid search. The class-wise AUCs of the four best-performing windows (Windows 1–4) and the baseline without windowing are reported. Additionally, mean validation AUCs are provided. The highest AUC values are highlighted in bold. Enlarged cardiom. = enlarged cardiomediastinum.

Window	None (Baseline)	#1	#2	#3	#4
Level	2048	2500	1750	2750	2250
Width	4096	3000	3000	3000	3000
**Finding**					
Atelectasis	0.749	0.756	0.753	0.749	**0.757**
Cardiomegaly	0.774	0.783	**0.786**	0.774	0.777
Consolidation	0.742	**0.744**	0.743	0.742	0.740
Edema	0.833	0.830	**0.841**	0.833	0.831
Enlarged cardiom.	0.701	**0.710**	0.700	0.701	0.686
Fracture	**0.710**	0.695	0.670	**0.710**	0.669
Lung lesion	0.682	**0.720**	0.710	0.682	0.700
Lung opacity	**0.690**	0.683	0.686	**0.690**	0.684
No finding	0.797	**0.804**	0.800	0.797	0.798
Pleural effusion	0.879	**0.888**	0.883	0.879	0.885
Pleural other	0.831	0.841	0.820	0.831	**0.850**
Pneumonia	**0.698**	0.686	0.690	**0.698**	0.683
Pneumothorax	0.828	0.822	**0.832**	0.828	0.809
Support devices	0.888	0.887	0.887	0.888	**0.889**
Mean (validation)	0.804	**0.807**	0.802	0.805	0.803
Mean (test)	0.772	**0.775**	0.772	0.772	0.768

**Table 4:** Comparison of baseline (8-bit), WindowNet without windowing (“No Windowing”) and with random contrast and brightness (“Augmentations”), and WindowNet AUCs for chest X-ray classification with 95 % confidence intervals. Higher values are highlighted in bold. Enlarged cardiom. = enlarged cardiomediastinum.

Finding	8-Bit	No Windowing	Augmentations	WindowNet
Atelectasis	0.751 [0.736–0.767]	0.812 [0.794–0.830]	0.824 [0.806–0.841]	**0.829** [0.811–0.846]
Cardiomegaly	0.770 [0.757–0.784]	0.814 [0.797–0.831]	0.826 [0.809–0.842]	**0.827** [0.810–0.843]
Consolidation	0.740 [0.715–0.765]	0.808 [0.773–0.841]	**0.828** [0.796–0.859]	0.823 [0.789–0.855]
Edema	0.831 [0.818–0.844]	0.891 [0.875–0.907]	0.892 [0.876–0.908]	**0.897** [0.880–0.912]
Enlarged cardiom.	0.691 [0.656–0.726]	0.745 [0.698–0.790]	0.746 [0.698–0.792]	**0.764** [0.715–0.812]
Fracture	**0.664** [0.624–0.705]	0.619 [0.525–0.711]	0.563 [0.469–0.658]	0.615 [0.517–0.709]
Lung lesion	0.680 [0.644–0.716]	0.701 [0.652–0.749]	**0.761** [0.711–0.808]	0.744 [0.691–0.793]
Lung opacity	0.680 [0.665–0.695]	0.726 [0.704–0.748]	**0.746** [0.724–0.768]	0.745 [0.724–0.766]
No finding	0.789 [0.774–0.805]	0.855 [0.841–0.869]	0.858 [0.844–0.872]	**0.859** [0.845–0.873]
Pleural effusion	0.883 [0.873–0.892]	0.909 [0.898–0.920]	0.915 [0.903–0.926]	**0.918** [0.907–0.928]
Pleural other	**0.823** [0.789–0.854]	0.721 [0.631–0.806]	0.803 [0.725–0.875]	0.793 [0.721–0.856]
Pneumonia	0.659 [0.634–0.684]	0.731 [0.694–0.765]	0.727 [0.691–0.762]	**0.750** [0.716–0.782]
Pneumothorax	0.802 [0.766–0.836]	0.830 [0.793–0.864]	0.856 [0.819–0.888]	**0.886** [0.856–0.913]
Support devices	0.868 [0.857–0.879]	0.897 [0.884–0.910]	0.909 [0.896–0.922]	**0.918** [0.906–0.930]
Mean	0.759	0.790	0.804	**0.812**

## Data Availability

The MIMIC data set used in this study is available at https://physionet.org/content/mimic-cxr/2.0.0/, last accessed on 1 December 2023.
